# IRD-dataset: a multi-source Iraqi road defect dataset for detection, segmentation, and GPS-based mapping

**DOI:** 10.1016/j.dib.2026.113250

**Published:** 2026-09-07

**Authors:** Zainab J. Ahmed, Hussein K. Khafaji

**Affiliations:** a Informatics Institute for Postgraduate Studies, University of Information Technology and Communications (UoITC), Baghdad, Iraq; b Department of Biology, College of Science, University of Baghdad, Baghdad, Iraq; c Communication Technologies Engineering Department, Technical Engineering College, Uruk University, Iraq

**Keywords:** Pavement condition assessment, Computer vision, Bounding box annotations, Polygon annotations, Semantic masks, Geolocation metadata, Urban infrastructure inspection

## Abstract

Road defect datasets support the development of computer vision methods for pavement inspection, road condition assessment, and maintenance planning. The public pavement defect datasets cited in this article are based on data collected outside Iraq. This article introduces IRD-Dataset, a public multi-source dataset documenting paved road environments in Baghdad, Iraq. The dataset consists of 4,352 RGB images collected from public paved road environments using three sources: Dashcam (1,006 images), Drone (2,162 images), and Mobile (1,184 images). Images were captured under varied field conditions and screened to retain those suitable for annotation and public release. Five road surface categories are included in the dataset: longitudinal crack, transverse crack, alligator crack, pothole, and speed bump. The first four categories are pavement defects, whereas speed bumps are intentional road features included for practical road-scene analysis. The images were manually annotated with bounding boxes for object detection and polygons for instance segmentation. The release also provides segmentation masks, image-level GPS metadata, 401 background images, and a YOLO configuration file. IRD-Dataset supports annotation-derived image classification, object detection, instance segmentation, semantic segmentation mask generation, and image-level GPS mapping of road surface observations. The data may support pavement monitoring, municipal road maintenance planning, and smart city road condition analysis.

Specifications Table

**Table:** 

Subject	Computer Sciences
Specific subject area	Annotation-derived image-level classification, multiclass road surface detection, instance segmentation, mask generation, and GPS-based road mapping.
Type of data	Filtered and privacy-processed 2D RGB road images (.jpg); processed YOLO bounding box (BBox) annotation files (.txt); processed YOLO polygon annotation files (.txt); processed SegmentationClass and SegmentationObject mask images (.png); tabular image-level GPS metadata (.csv); and YOLO configuration data (.yaml).
Data collection	Images were collected in Baghdad over approximately two winter months using an Abask 4K WiFi GPS Dashcam, a DJI Phantom 4 drone, and an iPhone 16 Pro Max. The initial collection contained approximately 16,000 images. Blurry, repeated or near-duplicate, very dark, motion-blurred, non-annotatable, or irrelevant non-road images were excluded. A total of 4,352 images were retained, including 401 background images. Target instances were manually annotated using CVAT.
Data source location	Baghdad, Iraq. Image-level latitude and longitude coordinates for the released images are provided in the accompanying GPS metadata CSV file.
Data accessibility	Repository name: Zenodo Data identification number: 10.5281/zenodo.21370736 Direct URL to data: 10.5281/zenodo.21370736 Instructions for accessing these data: Download all dataset files from Zenodo. The images folder is provided as five ZIP archives due to upload size limits: Dashcam, Drone part1, Drone part2, Mobile part1, and Mobile part2. Extract all ZIP files into the same IRD-Dataset root folder. If the extraction tool asks about an existing images folder, choose merge, not skip or replace, so that all image files are combined into one images/ folder. The labels_bbox, labels_polygon, masks, and gps_metadata folders are each provided as a single ZIP archive.
Related research article	None

## 1 Value of the Data

• IRD-Dataset provides a public multi-source Iraqi road image collection that enables cross-source evaluation across Dashcam, Drone, and Mobile viewpoints.
• Multiple vision tasks can be performed on a single image collection, with bounding box labels, polygon labels, SegmentationClass masks, and SegmentationObject masks.
• The image-level GPS metadata enable geographic mapping of road surface observations and integration with visualization and maintenance planning workflows.
• The 401 background images with empty YOLO annotation files can help researchers assess false-positive predictions on road scenes without target classes.
• The public release includes anonymized road images that can be reused in research and teaching.

## 2 Background

Image-based pavement monitoring has become an important methodological direction for road surface inspection, where computer vision and deep learning methods require structured image datasets with reliable annotations [[1], [2], [3]]. Existing public road defect and pavement crack datasets differ in geographic coverage, acquisition source, annotation format, and supported tasks [[4], [5], [6], [7], [8], [9]]. These datasets include vehicle-based, smartphone, close-range, and UAV pavement images with annotations supporting classification, object detection, and pixel-level analysis [[4], [5], [6], [7], [8], [9]]. The public datasets cited here do not include an Iraqi dataset that combines Dashcam, Drone, and Mobile imagery with image-level GPS metadata. IRD-Dataset was compiled to document paved road surface conditions in Baghdad, Iraq, under real-world field conditions and to organize the collected images as a reusable public data resource.

## 3 Data Description

IRD-Dataset is a multi-source collection for Iraqi paved road surface analysis, with images collected from public paved road environments in Baghdad, Iraq [10]. Table 1 summarizes the acquisition sources and image counts.

**Table 1:** Source-wise image distribution in the IRD-Dataset.

Source	Image count
Dashcam	1,006
Drone	2,162
Mobile	1,184
Total	4,352

**Fig. 1:**
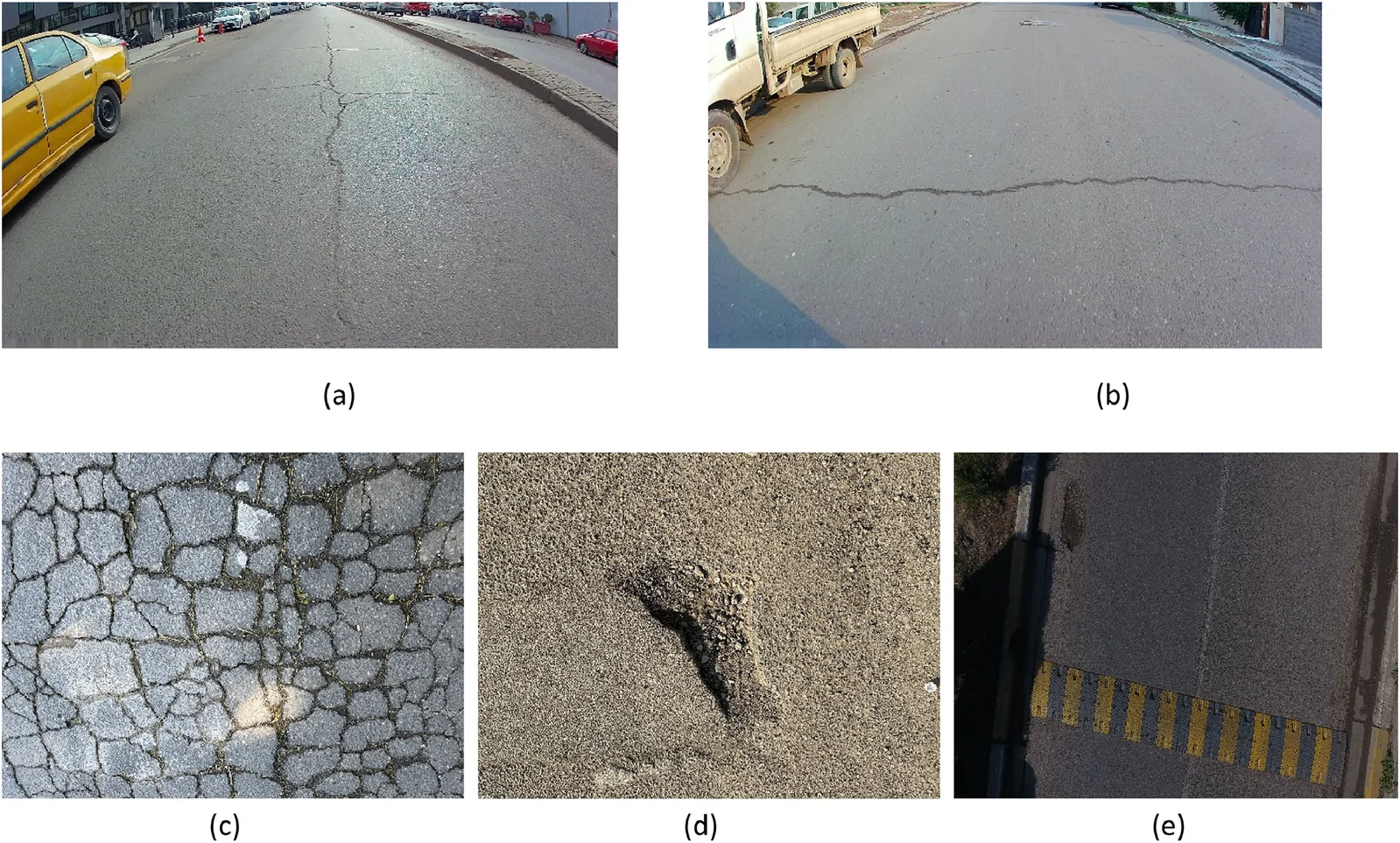
Representative samples of the five road surface classes in IRD-Dataset: (a) longitudinal crack; (b) transverse crack; (c) alligator crack; (d) pothole; and (e) speed bump.

The dataset covers five target classes, as described in Section 4.3. Representative examples of these classes are shown in Fig. 1. Background images without target classes were retained with empty YOLO annotation files and no segmentation masks. Segmentation masks are therefore provided for the 3,951 non-background images. No official training, validation, or test split is imposed in this data release, and the dataset contains no predefined training, validation, or test folders.

Table 2 presents the image-level class distribution for the bounding-box and polygon annotation versions.

**Table 2:** Image-level class distribution in the final IRD-Dataset.

Annotation	Total images	Background	D00	D10	D20	D40	SpeedBump
BBox	4,352	401	1,365	1,063	1,448	771	430
Polygon	4,352	401	1,365	1,063	1,448	771	430

Note: Image-level class counts are multi-label and may exceed the total number of images. The BBox and Polygon rows are identical because they report class presence in the same image set; object counts are reported separately in Table 3.

**Fig. 2:**
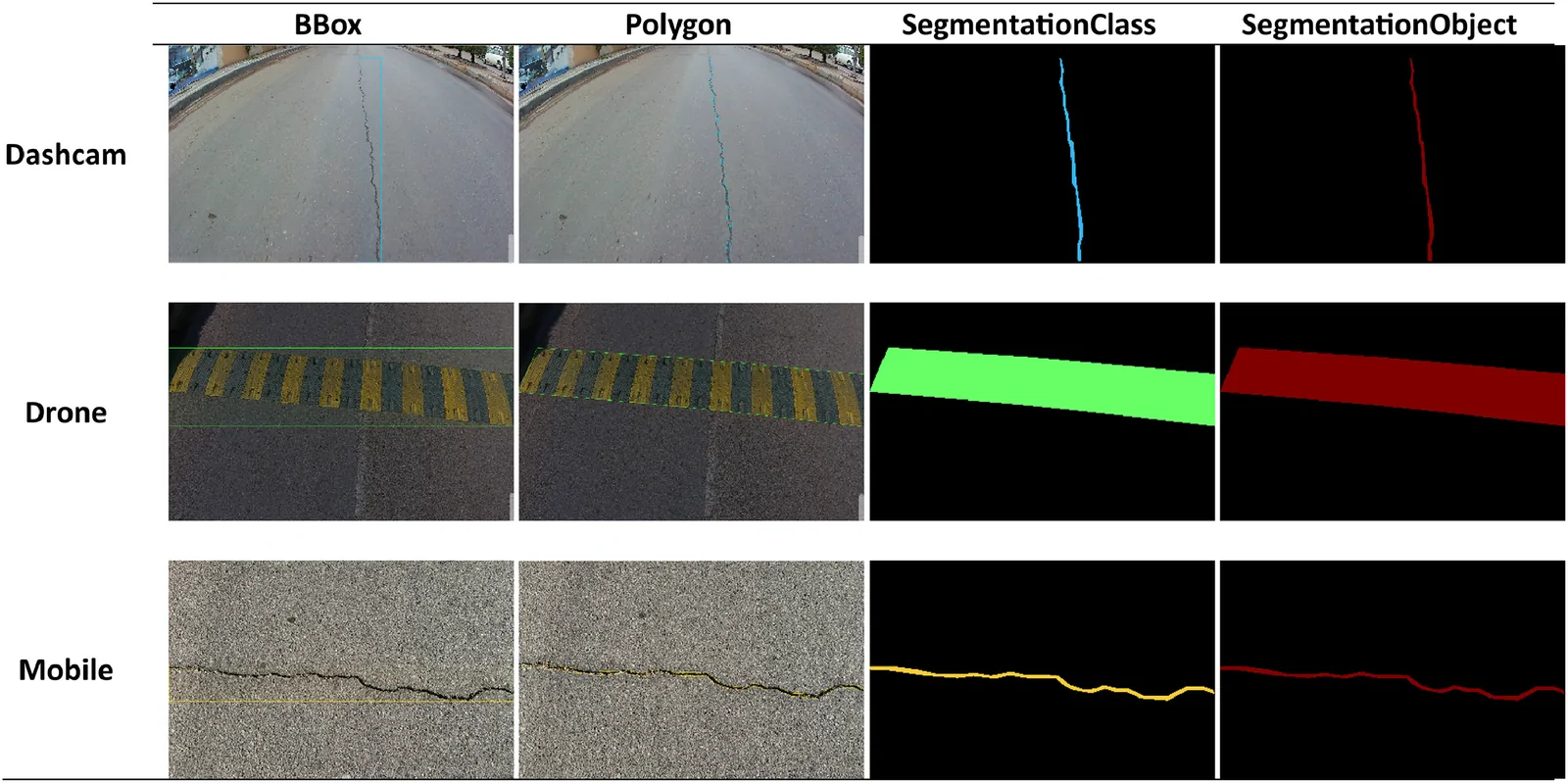
Examples of the released annotation formats. The rows represent Dashcam, Drone, and Mobile images, while the columns show bounding box annotations, polygon annotations for instance segmentation, SegmentationClass masks, and SegmentationObject masks.

Examples of the released annotation outputs, including bounding box annotations, polygon annotations, SegmentationClass masks, and SegmentationObject masks, are shown in Fig. 2.

Each bounding box annotation file uses one line per object in the format <class_id> <x_center> <y_center> <width> <height>. The bounding box coordinates are normalized relative to the image dimensions and range from 0 to 1 [11]. Each polygon annotation file also uses one line per object, beginning with the class identifier followed by a sequence of normalized vertex coordinates in the format <class_id> <x1> <y1> <x2> <y2> ... <xn> <yn>, which traces the object boundary [12].

Class identifiers begin at zero: 0 represents D00_LongitudinalCrack, 1 represents D10_TransverseCrack, 2 represents D20_AlligatorCrack, 3 represents D40_Pothole, and 4 represents SpeedBump. The SegmentationClass masks use fixed RGB colors defined in labelmap.txt: D00_LongitudinalCrack is represented by (50, 183, 250), D10_TransverseCrack by (255, 204, 51), D20_AlligatorCrack by (240, 120, 240), D40_Pothole by (9, 5, 29), and SpeedBump by (102, 255, 102). Background pixels are represented by black, with an RGB value of (0, 0, 0). CVAT generated the SegmentationObject masks automatically to distinguish separate annotated instances within each image [13]. The final dataset contains 7,293 bounding box objects and 6,959 polygon objects. Table 3 presents their distribution by source and class.

**Table 3:** Source-wise object counts by class for bounding box and polygon annotations.

Annotation	Source	D00	D10	D20	D40	SpeedBump	Total Objects
BBox	Dashcam	1,017	350	64	174	0	1,605
BBox	Drone	685	827	2,049	473	178	4,212
BBox	Mobile	272	374	211	256	363	1,476
Polygon	Dashcam	982	366	64	174	0	1,586
Polygon	Drone	635	844	1,724	474	185	3,862
Polygon	Mobile	282	373	211	256	389	1,511

The released repository contains road images in images/, bounding box annotations in labels_bbox/, polygon annotations in labels_polygon/, and segmentation masks in masks/. The masks/ folder contains SegmentationClass/, SegmentationObject/, and labelmap.txt. The gps_metadata/ folder contains IRD_Dataset_GPS_Metadata.csv, which provides the image-level GPS metadata.

The road images are provided as .jpg files, while bounding box and polygon annotations are stored as .txt files. The SegmentationClass and SegmentationObject folders each contain 3,951 .png mask images. Table 4 describes the columns contained in the GPS metadata file.

**Table 4:** Description of the GPS metadata columns.

Column	Meaning
source	Acquisition source: Dash (Dashcam), Drone, or Mobile
image_name	Name of the corresponding image file
label_name	Name of the corresponding annotation file
lat	Latitude in decimal degrees
lon	Longitude in decimal degrees
alt_m	Altitude in metres, mainly available for Drone images
t_sec	Temporal position of a Dashcam image within its original video, measured in seconds from the beginning of that video
image_path	Relative path to the image file
bbox_label_path	Relative path to the bounding-box annotation file
polygon_label_path	Relative path to the polygon annotation file
segmentation_class_path	Relative path to the SegmentationClass mask
segmentation_object_path	Relative path to the SegmentationObject mask
mask_path	Relative path to the segmentation object mask when available
has_mask	Boolean value indicating whether segmentation masks are available

Fields that do not apply to a particular acquisition source are left empty. The mask_path field duplicates segmentation_object_path when masks are available. Relative paths are used to maintain dataset portability across computer systems. The repository includes several supporting files. The data.yaml file defines the dataset folder paths and the five class identifiers and their names. The labelmap.txt file defines the RGB colors used in the SegmentationClass masks, including black for background pixels. The README.md file provides the dataset description, folder structure, annotation formats, usage guidance, privacy information, limitations, licensing details, and contact information. The CITATION.cff file provides the dataset authors, version, DOI, license, and recommended citation metadata. The checksums_sha256.txt file contains SHA-256 hash values for the extracted files and can be used to verify file integrity after download and extraction. The LICENSE.txt file specifies the CC BY 4.0 reuse and attribution conditions.

**Fig. 3:**
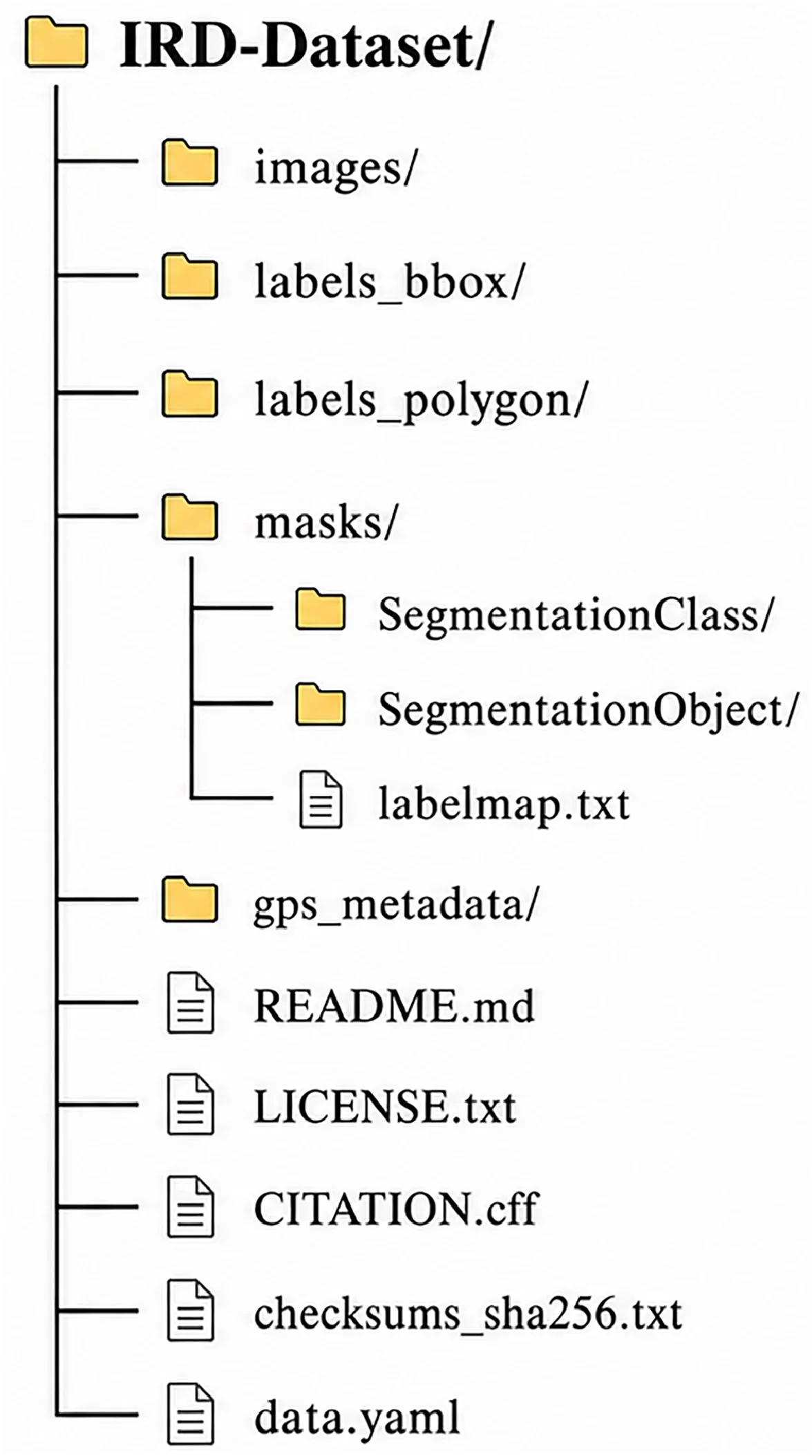
Public folder structure of IRD-Dataset.

On Zenodo, the image data are distributed across five ZIP archives: IRD-Dataset_v1.0.0_images_Dashcam.zip, IRD-Dataset_v1.0.0_images_Drone_part1.zip, IRD-Dataset_v1.0.0_images_Drone_part2.zip, IRD-Dataset_v1.0.0_images_Mobile_part1.zip, and IRD-Dataset_v1.0.0_images_Mobile_part2.zip. Additional archives include the following:

Bounding box labels (IRD-Dataset_v1.0.0_labels_bbox.zip), polygon labels (IRD-Dataset_v1.0.0_labels_polygon.zip), segmentation masks (IRD-Dataset_v1.0.0_masks.zip), and GPS metadata (IRD-Dataset_v1.0.0_gps_metadata.zip). After extraction, these archives form the folder structure shown in Fig. 3.

**Fig. 4:**
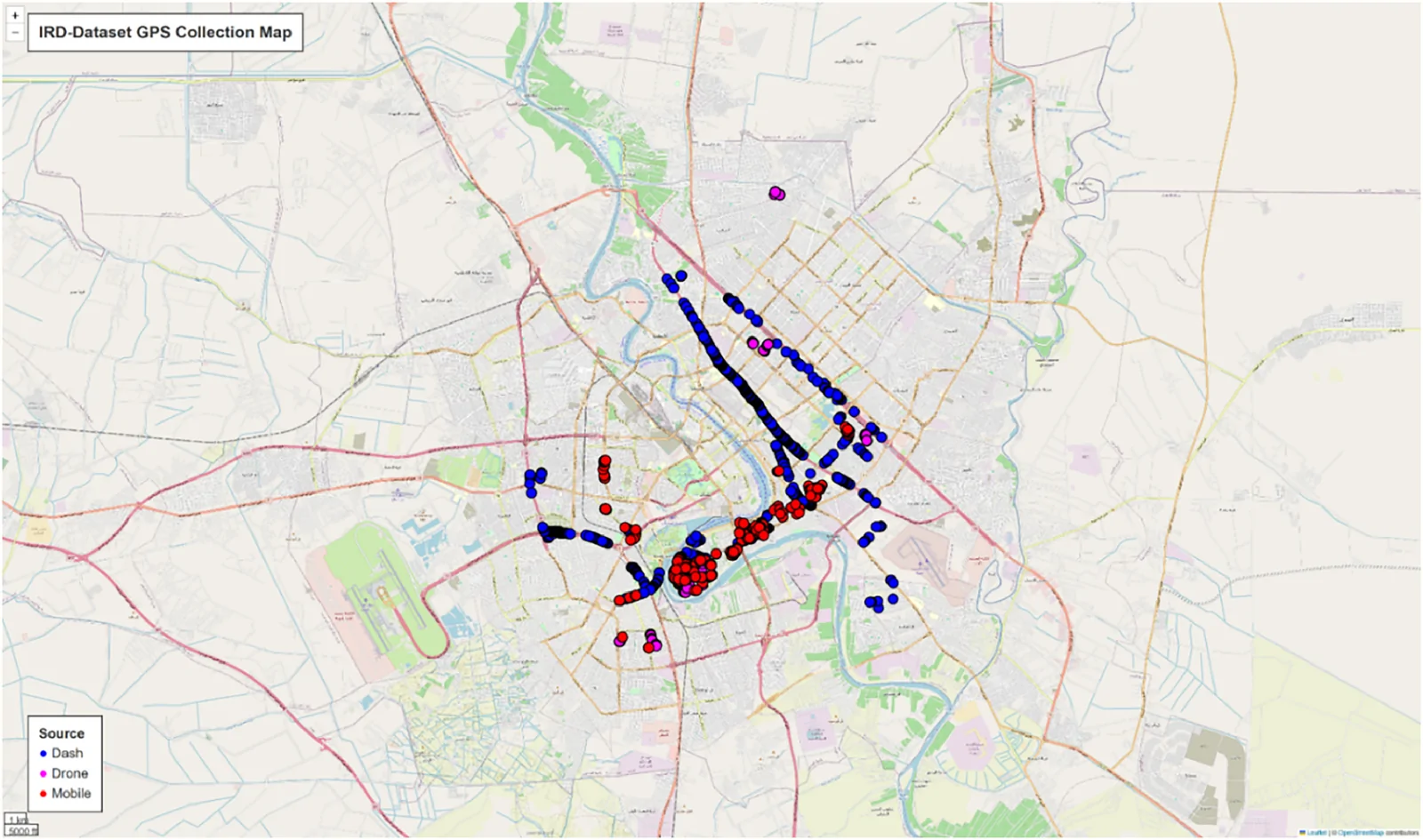
Spatial distribution of the released IRD-Dataset images in Baghdad, Iraq.

Fig. 4 shows the spatial distribution of the released IRD-Dataset images in Baghdad, Iraq, based on GPS coordinates associated with the Dashcam, Drone, and Mobile acquisition sources.

## 4 Experimental Design, Materials and Methods

### 4.1 Data Collection Design

IRD-Dataset images were acquired from public paved road environments in Baghdad, Iraq, using a Drone, Dashcam, and Mobile cameras. Data collection was conducted over approximately two months during winter. Images were acquired under different visual conditions, including sunny, cloudy, shadowed, low-light, and post-rain conditions.

**Fig. 5:**
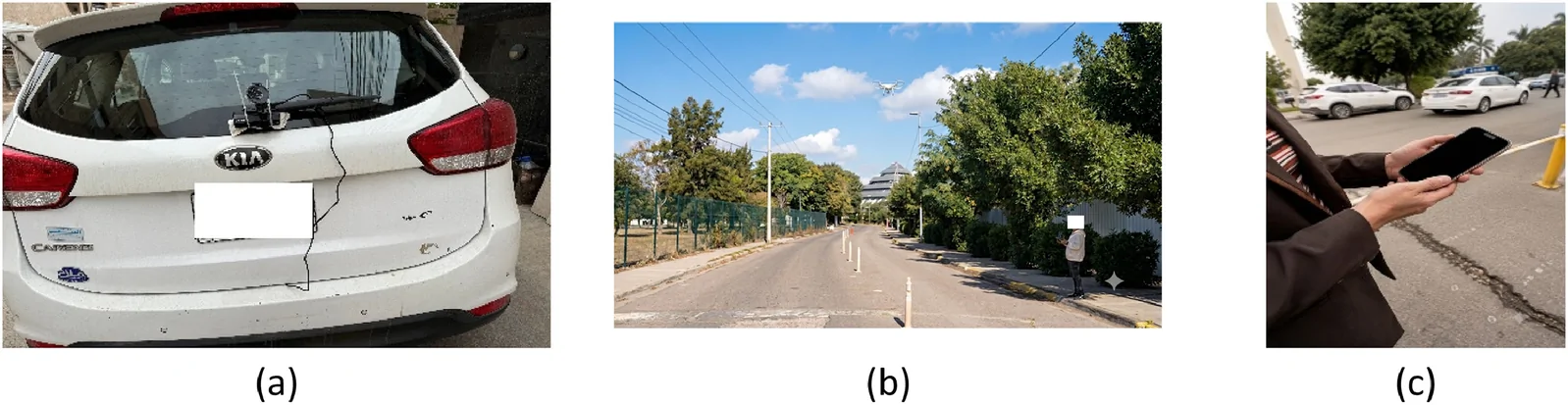
Examples of the three image acquisition setups used for IRD-Dataset: (a) rear-mounted vehicle Dashcam; (b) Drone-based road imaging; and (c) Mobile camera acquisition.

Drone photographs were taken with a DJI Phantom 4 at a resolution of 4000 × 2250 pixels. Gimbal pitch varied across the collection; most views were close to nadir, with pitch angles of about 80° to 90°, while some oblique views were captured at about 35° to 45°. Oblique images were kept when the road surface and target boundaries remained clear enough for reliable annotation. Flight height was generally about 4–8 m above the road and was adjusted when required by wind, obstacles, trees, power lines, or passing vehicles. Permission for drone photography was obtained. Dashcam imagery was recorded at 3840 × 2160 pixels with an Abask 4K Wi-Fi GPS Dashcam mounted on a vehicle about 1 m above the ground at an angle of approximately 45°. Vehicle speed was generally between 30 and 50 km/h, depending on traffic and safety conditions. The video files were about one minute long. One frame was sampled every second using Python and OpenCV (cv2.VideoCapture), with cv2.CAP_PROP_POS_MSEC was used to access the corresponding video time position. This yielded about 60 sampled frames per clip. The sampled frames were initially retained and later reviewed as described in Section 4.2. Mobile photographs were taken with an iPhone 16 Pro Max in Most Compatible JPEG mode at a resolution of 4032 × 3024 pixels. The camera was positioned approximately 1 m above the road surface or slightly lower and was oriented either perpendicular to the target area or at a slight angle. Fig. 5 shows representative examples of the three acquisition sources used for IRD-Dataset image collection.

### 4.2 Image Filtering and Final Selection

The raw image collection contained about 16,000 images. During screening, images unsuitable for public release or annotation were removed. These included blurred, duplicate or near-duplicate, very dark, or severely motion-blurred images; images lacking useful road surface information; images with unclear or non-annotatable targets; views whose acquisition angle prevented reliable identification of the road surface or target boundaries; and irrelevant non-road scenes. Road images containing no target class were retained as background samples with empty YOLO annotation files. Following screening, 4,352 images remained, including 401 background images.

### 4.3 Road Surface Classes and Annotation Protocol

Five categories were used to describe paved road surfaces: D00_LongitudinalCrack, D10_TransverseCrack, D20_AlligatorCrack, D40_Pothole, and SpeedBump. The four defect categories represent road damage, whereas SpeedBump is an intentional road feature included for practical road-scene analysis. CVAT, deployed locally through Docker in an Ubuntu environment, was used to manually annotate the images [13]. The full dataset was manually annotated by one primary annotator. For independent quality assessment, a second annotator independently re-annotated 220 images, representing 5.1% of the dataset. The manual annotations form the basis of the released labels and masks. The same class definitions were applied to the bounding box and polygon annotations. Bounding boxes were prepared for object-detection tasks, while polygons were prepared for instance-segmentation tasks. The polygon annotations were also exported using the CVAT Segmentation Mask 1.1 format to generate SegmentationClass and SegmentationObject masks automatically. The SegmentationClass masks provide class-level segmentation information, whereas the SegmentationObject masks distinguish individual object instances. The annotation files were exported in Ultralytics YOLO Detection 1.0 and Ultralytics YOLO Segmentation 1.0 formats. Pavement seams and construction joints were not annotated as road defects. Only road surface targets with sufficiently clear and visible boundaries were annotated.

### 4.4 GPS Metadata Preparation

Each image was associated with GPS metadata. Drone and Mobile images contained embedded location information, while Dashcam GPS data were recorded by the built-in GPS module of the Abask dashcam. Drone coordinates were extracted from the embedded image metadata. Dashcam GPS logs were exported using Viidure GPS Player to obtain latitude and longitude information. Mobile coordinates were extracted from the image EXIF metadata because location services, camera location access, and precise location were enabled on the iPhone.

Metadata from the three acquisition sources were combined into a single CSV file. The spatial distribution map was generated using Folium [14]. For Dashcam images, t_sec records elapsed time from the beginning of each video clip and restarts for each new clip.

### 4.5 Privacy Preprocessing and Public-Release Preparation

**Fig. 6:**
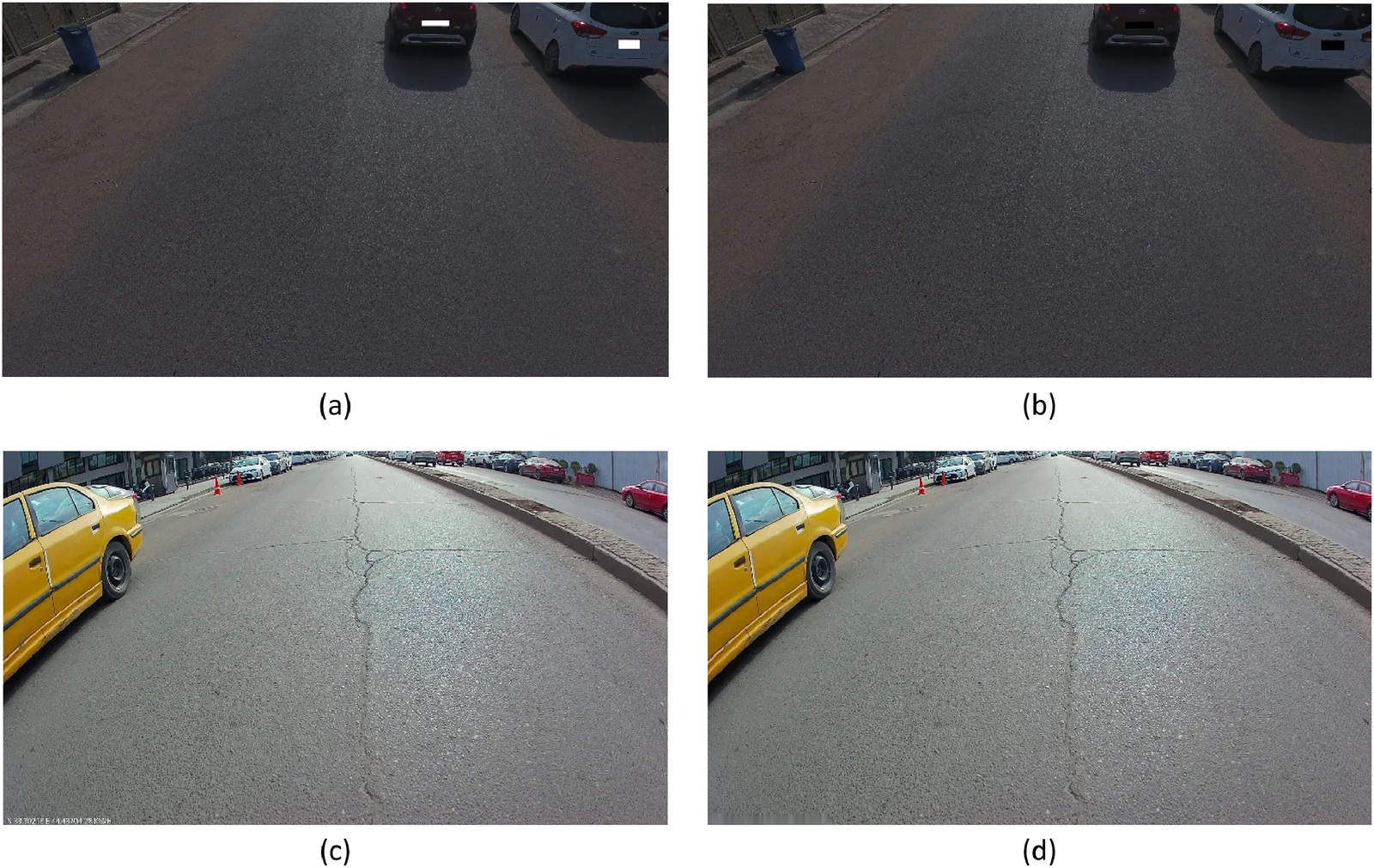
Examples of privacy anonymization and Dashcam GPS overlay removal: (a) Drone source image before final anonymization for public release, with identifiers redacted in the manuscript; (b) Drone image after face and license plate masking; (c) Dashcam source image before GPS overlay removal, with identifiers redacted in the manuscript; and (d) Dashcam image after GPS overlay removal and license plate masking.

Privacy-related preprocessing was applied before public release. Dashcam images contained a visible GPS overlay in the lower-left corner. A fixed 720 × 60-pixel region was masked, and the overlay was removed using Telea inpainting [15]. The region boundaries were defined as x_1_ = 0, x_2_ = min (720, W), y_1_ = max (0, H − 60), and y_2_ = H, where W and H are the image width and height, respectively. The operation was implemented in OpenCV using cv2.inpaint with an inpainting radius of 3 pixels and the cv2.INPAINT_TELEA flag. Solid black rectangular masks were used to anonymize visible human faces and vehicle license plates in Dashcam and Drone images. No visible identifiers requiring anonymization were present in the Mobile images. These preprocessing operations were limited to identifiable visual information and were not applied to the annotated road surface regions. The image dimensions, annotation files, and GPS metadata were preserved. Fig. 6 shows examples of the anonymization and GPS overlay removal procedures.

### 4.6 Technical Validation and Data Quality Control

Data quality control was carried out during image selection, annotation, and preparation for public release. Image quality screening and exclusion were performed as described in Section 4.2. Automated checks were used to verify the correspondence between images and their bounding box and polygon annotation files. The annotation files were also checked for valid class identifiers, correct formatting, and normalized coordinates within the range of 0 to 1. No invalid bounding box or polygon annotation files were identified. These checks evaluated file structure, class identifiers, and coordinate validity. The object totals obtained from these checks were consistent with those reported in Table 3. File and annotation counts were cross-checked, together with the correspondence among image names, annotation names, mask files, and GPS metadata records. SHA-256 hashes were then generated for the extracted dataset files and recorded in checksums_sha256.txt, enabling users to check file integrity after download and extraction. The extracted Zenodo dataset was checked against the folder structure shown in Fig. 3. The SHA-256 checksums were verified against the downloaded files, with no missing files or checksum mismatches.

Inter-annotator consistency was assessed on 220 randomly selected images, equivalent to 5.1% of the dataset. A second annotator independently annotated this subset. Mean Intersection over Union (IoU) was 0.828 for bounding boxes and 0.770 for polygons. A third senior reviewer examined cases with ambiguous boundaries or lower spatial agreement and made the final annotation decision.

## Limitations

IRD-Dataset covers Baghdad only and reflects a two-month winter collection period. It therefore does not represent other Iraqi regions or seasonal conditions such as summer heat and heavy dust. The three acquisition sources are not equally represented, with more Drone images than Dashcam or Mobile images. Collection was also influenced by motion blur, traffic, occlusions, perspective distortion, shadows, camera vibration, flight conditions, and safety constraints. Class distributions are uneven across sources; Dashcam images contain no SpeedBump instances, and some source–class combinations, such as D20_AlligatorCrack in Dashcam images, contain relatively few objects. When creating training, validation, and test subsets, users should consider stratifying the data by acquisition source and class presence to reduce imbalance among the subsets. Training-only data augmentation may also be applied to underrepresented classes when needed. The dataset does not include engineering severity measurements such as crack or pothole depth, affected area, or repair priority. GPS coordinates are provided at the image level rather than for individual objects. No predefined training, validation, or test split is provided.

## Ethics Statement

The authors have read and followed the ethical requirements for publication in Data in Brief and confirm that the current work did not involve human subjects as research participants, animal experiments, or data collected from social media platforms. Visible human faces and vehicle license plates incidentally captured in the road images were anonymized before public release using solid black masking.

## CRediT Author Statement

**Zainab J. Ahmed:** Conceptualization, Methodology, Software, Validation, Investigation, Resources, Data curation, Visualization, Writing – original draft, Writing – review & editing. **Hussein K. Khafaji:** Supervision, Methodology, Validation, Writing – review & editing.

## Declaration of Generative Ai and Ai-Assisted Technologies in the Manuscript Preparation Process

During manuscript preparation, the authors used ChatGPT (OpenAI) and Claude (Anthropic) for language editing and text organization. All outputs were reviewed and verified by the authors, who take full responsibility for the final content.
